# *Glycyrrhiza uralensis* polysaccharides ameliorates cecal ligation and puncture-induced sepsis by inhibiting the cGAS-STING signaling pathway

**DOI:** 10.3389/fphar.2024.1374179

**Published:** 2024-06-05

**Authors:** Siwen Hui, Wen Kan, Shuanglin Qin, Ping He, Jia Zhao, Hui Li, Jun Bai, Jincai Wen, Wenqing Mou, Manting Hou, Ziying Wei, Li Lin, Xiaohe Xiao, Guang Xu, Zhaofang Bai

**Affiliations:** ^1^ Department of Hepatology, The Fifth Medical Center of Chinese PLA General Hospital, Beijing, China; ^2^ China Military Institute of Chinese Materia, Fifth Medical Center of Chinese PLA General Hospital, Beijing, China; ^3^ School of Pharmacy, Xianning Medical College, Hubei University of Science and Technology, Xianning, China; ^4^ Department of Neurosurgery, General Hospital of Chinese People Liberty Army, Beijing, China; ^5^ School of Traditional Chinese Medicine, Capital Medical University, Beijing, China

**Keywords:** *Glycyrrhiza uralensis* fisch., *Glycyrrhiza uralensis* polysaccharides, cGAS-STING pathway, sepsis, cecal ligation

## Abstract

**Ethnopharmacological relevance:** *G. uralensis Fisch.* (*Glycyrrhiza uralensis*) is an ancient and widely used traditional Chinese medicine with good efficacy in clearing heat and detoxifying action. Studies suggest that *Glycyrrhiza Uralensis* Polysaccharides (GUP), one of the major components of *G. uralensis,* has anti-inflammatory, anti-cancer and hepatoprotective effects., but its exact molecular mechanism has not been explored in depth.

**Aim of the study:** Objectives of our research are about exploring the anti-inflammatory role of GUP and the mechanisms of its action.

**Materials and methods:** ELISA kits, Western blotting, immunofluorescence, quantitative real-time PCR, immunoprecipitation and DMXAA-mediated STING activation mice models were performed to investigate the role of GUP on the cGAS-STING pathway. To determine the anti-inflammatory effects of GUP, cecal ligation and puncture (CLP) sepsis models were employed.

**Results:** GUP could effectively inhibit the activation of the cGAS-STING signaling pathway accompany by a decrease the expression of type I interferon-related genes and inflammatory factors in BMDMs, THP-1, and human PBMCs. Mechanistically, GUP does not affect the oligomerization of STING, but affects the interaction of STING with TBK1 and TBK1 with IRF3. Significantly, GUP had great therapeutic effects on DMXAA-induced agonist experiments *in vivo* as well as CLP sepsis in mice.

**Conclusion:** Our studies suggest that GUP is an effective inhibitor of the cGAS-STING pathway, which may be a potential medicine for the treatment of inflammatory diseases mediated by the cGAS-STING pathway.

## Graphical Abstract

**Figure:**
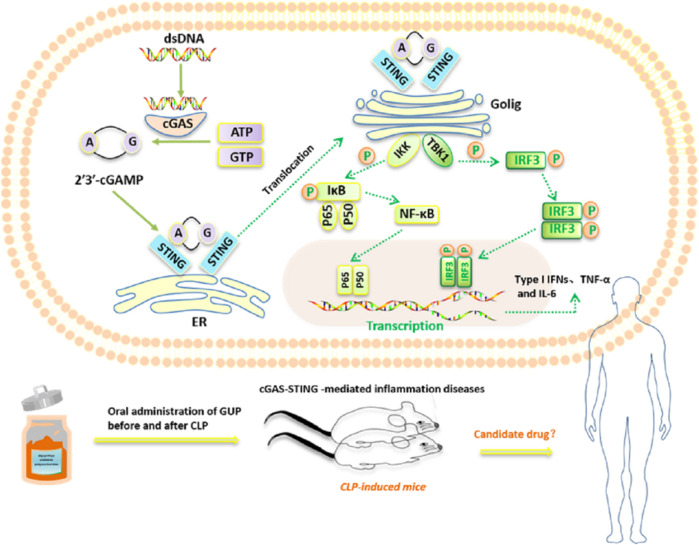
GUP can effectively inhibit the activation of cGAS-STING pathway induced by multiple stimuli and reduce the expression of type I interferon and pro-inflammatory cytokines. Meanwhile GUP also has a good therapeutic effect on sepsis contributed to cecum ligation and puncture.

## 1 Introduction

Sepsis is a systemic inflammatory response caused by bacterial or fungal infection; life-threatening multiple organ dysfunction developed as the disease progresses (Singer et al., 2016). Septic shock and sepsis are serious health problems that affect millions of people worldwide every year. They rank among the top ten leading causes of death and morbidity worldwide (Cheng et al., 2015). Despite excellent success achieved in early detection and emergency response management, sepsis remains an important factor in the death of infected patients (Fleischmann-Struzek et al., 2020). The CLP mice model has become the most widely used model for experimental sepsis and is now considered the gold standard for sepsis research (Dejager et al., 2011; Zoulikha et al., 2022). At the onset of sepsis, a systemic inflammatory response occurs, followed by septic shock, multi-organ dysfunction, and ultimately death. In the CLP mice model, multiple foci of microbial infection occur in the abdominal cavity, subsequently triggering a systemic inflammatory response. Studies have shown that ligation of the mouse cecum results in elevated levels of apoptosis and increased secretion of pro-inflammatory cytokines and interferon in the mouse intestinal epithelium (Buras et al., 2005). The search for precursor compounds that may be effective against sepsis by studying models of cecum ligation is an important approach to the clinical treatment of sepsis.

Recent studies indicate that the cGAS-STING pathway is an important immune response pathway in the organism and plays an important role in the regulation of pathological states such as infection and inflammation. The process of activation of the cGAS-STING pathway is associated with abnormal release of DNA from organisms infected with viruses, bacteria, parasites or intracellular DNA. The cytoplasmic pattern recognition receptor cGAS can be activated by exogenous DNA, DNA released by damaged cells and mitochondrial leakage of DNA as well as by intracellularly production of micronuclei, catalyzing the creation of second messenger cGAMP and stimulating STING signaling (Wu et al., 2013; Decout et al., 2021). After leaving the endoplasmic reticulum, STING moves to the Golgi apparatus and eventually to the perinuclear endosome, where it interacts with TNBK-binding kinase-1 (TBK1) (Kwon and Bakhoum, 2020). TBK1 then phosphorylates STING, IRF3 and TRAF6, as well as its self, leading to the release of type I interferon and other inflammatory cytokines (Wu et al., 2013; Frémond and Crow, 2021). A increasing amount of researches have shown that the immoderate activation of cGAS-STING pathway could trigger a range of inflammatory responses and autoimmune diseases, including alcoholic liver disease (Chen R. et al., 2021), ischemic brain injury (Li et al., 2023) and rheumatoid arthritis (Wang J. et al., 2019). Studies indicate that aberrant activation of the cGAS-STING pathway plays an important role in the onset and progression of sepsis, and that during the occurrence of an infection, bacterial viruses or other pathogens activate the cGAS-STING pathway, which triggers the production of intracellular signals including interferon and other inflammatory signals. (Li J. et al., 2022). Targeted modulation of the cGAS-STING pathway may be an important strategy for the treatment of sepsis.

*Glycyrrhiza uralensis* Fisch. (*Glycyrrhiza uralensis*) is an ancient and widely-used traditional Chinese medicine, with the effects of tonifying the spleen, benefiting the qi, clearing heat and detoxifying the toxin, which has been recorded in many national pharmacopoeias (Jiang et al., 2022; Liu et al., 2023). In addition, *G. uralensis* has such functions as anti-cancer (Luo et al., 2021), anti-inflammatory (Li Q. et al., 2022; Sun et al., 2022), liver protection (Rizzato et al., 2017) and other pharmacological activities (Wahab et al., 2021). The manifestation of these pharmacological effects is mainly dependent on the chemical composition of *G. uralensis*. Studies have confirmed that Glycyrrhizic acid suppresses inflammatory reaction by inhibiting NF-κB and MAPK pathways, which has a protective effect against sepsis-induced acute lung injury (Gu et al., 2022; Yang et al., 9900). Glycyrrhizin has been shown to inhibit activation of the HMGB1/TLR9 pathway to alleviate sepsis-induced acute respiratory distress syndrome (Zhao et al., 2016). In addition, licorice flavonoids, licorice extracts, Licochalcone B, and Glabridin have all been shown to have significant inhibitory effects on the cGAS-STING pathway and its mediated inflammatory disorders (Wen et al., 2023a; Wen et al., 2023b; Luo et al., 2023; Luo et al., 2024). Therefore, we speculate that GUP, a major component of licorice, may also have an effect on the cGAS-STING pathway. Recent studies also depicted that polysaccharides and other *G. uralensis* extracts are the main active components with potent biological and pharmacological activities (Pastorino et al., 2018). Polysaccharides have greater development value and application potential in anti-inflammatory. For example, astragalus polysaccharides reduces gouty arthritis (Ren L. et al., 2022), lentinan has a good inhibitory effect on neuroinflammation (Zheng et al., 2005). As one of the main active ingredients of *G. uralensis*, GUP has not only enhanced immune regulation, anti-tumor and antioxidant effects (Chen et al., 2013; Nosalova et al., 2013; Lian et al., 2018), but also exhibit anti-inflammatory effects (Huang C. et al., 2022). Although the anti-inflammatory activity of GUP has been established, the exact mechanism of action and molecular targets remain unclear.

In our research, we aimed to explore the effect of GUP in the treatment of inflammation, particularly in the cGAS-STING pathway-mediated CLP sepsis, and to investigate the underlying mechanisms of its therapeutic role. We found that GUP inhibited the activation of the cGAS-STING pathway by inhibiting the interaction of STING with TBK1 and TBK1 with IRF3, thereby reducing the harm of sepsis.

## 2 Methods and materials

### 2.1 Reagents

GUP (WH-0960) was gained from Shanghai Ronghe Medical Technology Development Co., LTD. Its purity was ≥95.0% (purity values are presented by the company) and the original materials of GUP (*G. uralensis*-20210,824-1) was gained from Shanghai Wanshicheng Sinopod Products Co., LTD. Anti-α-tubulin (66031-1-Ig, PTG), anti-Lamin B1 (66095-1-Ig, PTG), anti-IRF3 (11312-1-AP, PTG), anti-HSP90 (13171-1-AP, PTG), anti-hp-IRF3 (ab76493, abcam), anti-STING (19851-1-AP, PTG), anti-mp-IRF3 (GTX86691, GeneTex), anti-HA Tag (66006-2-Ig, PTG), anti-DYKDDDDK tag Polyclonal (20543-1-AP, PTG), HT-DNA (D6898, Sigma), 2′3′-cGAMP (HY-100564A, MCE), diABZI (HY112921B, MCE), DMXAA (HY-10964, MCE), CCK-8 (KTA1020-1000T), Hoechst (Boston, United States), opti-MEM (2427634, Gibco), Protease inhibitors Cocktail (C0001, TargetMol).

The process of GUP extraction: The *G. uralensis* would be washed, dried, sliced, and then *G. uralensis* with distilled water was put in a vessel, heated, and kept 80°C for 2 h and stirred regularly. The filtrates were combined and concentrated, extracted three times with ethyl acetate, recovered the aqueous layer and discarded the ethyl acetate. Subsequently, the water layer was placed in AB-8 microporous adsorbent resin, washed with distilled water and recycled water parts. Finally, the recovered water part was dried in a freeze dryer for 48 h to access purified GUP (company supplied).

### 2.2 Animals

C57BL/6 mice (female, 8 weeks old) were purchased from SPF Biotechnology Co., Ltd. (Beijing, China). Mouse cages were kept pathogen-free (SPF) and provided unrestricted access to food and water. The ambient humidity is 50% ± 5% and the temperature is 20°C–22°C. We try our best to minimize the number of experimental animals and the least possible suffering of mice during the experiment. The Fifth Medical Center of the Chinese People’s Liberation Army General Hospital approved all animal experimental procedures used in this study. These procedures were all carried out in accordance with guidelines for the care and use of laboratory animals (IACUC-2021-0008).

### 2.3 The quality control of GUP

We employed Thermo Fisher ICS5000 ion chromatography to analyze the monosaccharide content of GUP. The chromatographic column used is DionexCarbopac^TM^PA20 (3*150). The chromatographic mobile phase: solvent A, H_2_O and solvent B, 15 mM NaOH and solvent C, 15 mM NaOH and 100 mM NaOAC. The flow rate was 0.3 mL/min, the injection volume was 5 μL, and the column temperature was set at 30°C. 16 monosaccharide standards including glucose, galactose and glucuronide were dissolved as controls. In the ampoule, the 10 mg sample was precisely weighed before 2 mL of 3M TFA was inserted and the sample was hydrolyzed at 120°C for 3 h. The acid hydrolysate was sucked out and blown dry with nitrogen. Add 5 mL of water vortexed to mix and draw 50 µL from it, add 950 µL of deionized water and centrifuge. Then, it is tested on an ion chromatograph.

### 2.4 Cell culture

Mouse bone marrow-derived macrophages (BMDMs) was isolated from bone marrow of 10-week-old female mice and incubated with DMEM medium containing murine macrophage colony-stimulating factor (MCE, Mon-mouth, NJ) with the addition of 1% penicillin/streptomycin (Macgene) and 10% fetal bovine serum (Gibco, Rockford, IL). Human primary monocytes (THP-1) were obtained from ATCC and cultured in RPMI 1640 medium. HEK-293T cells were provided by Tao Li (National Center of Biomedical Analysis) and was cultured in DMEM medium. Healthy volunteers provided the human PBMCs, which were cultivated in RPMI 1640 medium. The cell lines were developed at 37°C in an incubator with 5% CO_2_ humidity. Human Ethics Number is KY-2023-2-10-1.

### 2.5 Cell viability assay

This experiment adopted the Cell Counting Kit 8 (CCK-8) to detect cell viability. The cells were exposed to varying amounts of GUP for a duration of 12 h. CCK-8 reagent was co-incubated with the cells in the incubator for a duration of 1 h. Finally, optical density values (O.D.) at 450 nm were measured. Based on the results of the CCK8 toxicity test for GUP, subsequent experiments were conducted at a non-toxic dose of GUP.

### 2.6 Activation of the cGAS-STING pathway

THP-1 cells (with PMA), human PBMCs, BMDMs and were inoculated overnight in well plates respectively at a density of 1.3 × 10^6^, 2.5 × 10^6^ and 1.2 × 10^6^ cells/mL. Next day, GUP was dissolved in opti-MEM and filtered through a non-pyrogenic filter membrane with a pore size of 0.22 μM. The cells were then treated with GUP. After 1 h, transfection of HT-DNA (2.5 μg/mL) or 2′3′-cGAMP (2 μg/mL), or addition of diABZI (10 mmol/mL) or DMXAA (2.5 μg/mL) was carried out in the cells. For Western blot analysis, we collected cell lysates after 2 h. After 4 h, lysates from cells lysed with TRIZOL were collected for qPCR.

### 2.7 ELISA

According to the instructions, serum and intraperitoneal lavage liquid of mice were tested by mouse IL-6 (1210602, Dakewe), mouse TNF-α (1217202, Dakewe) and IFN-β (luex-mifnbv2, InvivoGen).

### 2.8 Western blotting

The conventional method of protein extraction was used (Wang Z. et al., 2019). Cell supernatant was collected, and centrifuged for 5 min, and whole cell lysate was prepared using 1× RIPA buffer. The supernatant of the centrifuged cell supernatant was then discarded and the fully lysed cell lysate was added. 10% SDS-PAGE was used to separate the protein samples at 100 V, and wet transfer was used to transfer the gels onto PVDF membranes. The PVDF membrane was then closed at room temperature in 10% skimmed milk for 1 h. We incubated PVDF overnight at 4°C with primary antibody, followed by 1 h with matched secondary antibody. Washing with TBST was required between each step. Detection of protein bands used enhanced chemiluminescence reagents (Millipore, Massachusetts, United States).

### 2.9 Reverse transcriptase quantitative PCR

Total RNA was collected using the TRIZOL reagent (Sigma, 93289) following the guidelines provided by the manufacturer. The RT Master Mix was utilized to transcribe cDNA from RNA in equal quantities (A230, GenStar). The SYBR Green qPCR Master Mix (HY-K0501, MCE) was utilized for conducting real-time PCR. Table 1 and Table 2 display the primer sequences.

**TABLE 1:** Mouse primers for quantitative real-time PCR analysis.

Target gene	Sequence (5′–3′)
Mouse TNF-α-	GGG​CAG​TTA​GGC​ATG​GGA​T
	TGA​GCC​TTT​TAG​GCT​TCC​CAG
Mouse IFN-β	TCC​GAG​CAG​AGA​TCT​TCA​GGA​A
	TGCAAC CACCACTCATTCTGAG
Mouse CXCL10	ATCATCCCTG CGAGCCTATCCT
	GAC​CTT​TTT​TGG​CTA​AAC​GCT​TTC
Mouse IL-6	CAC​TTC​ACA​AGT​CGG​AGG​CT
	CTG​CAA​GTG​CAT​CAT​CGT​TGT
Mouse GAPDH	CGG​AGT​CAA​CGG​ATT​TGG​TC
	GAC​AAG​CTT​CCC​GTT​CTC​AG
Mouse Actin	GGC​TGT​ATT​CCC​CTC​CAT​CG
	CCA​GTT​GGT​AAC​AAT​GCC​ATG​T

**TABLE 2:** Human primers for quantitative real-time PCR analysis.

Target gene	Sequence (5′–3′)
Human TNF-α-	CCT​CTC​TCT​AAT​CAG​CCC​TCT​G
	GAG​GAC​CTG​GGA​GTA​GAT​GAG
Human IFN-β	TCC​AAA​TTG​CTC​TCC​TGT​TG
	GCA​GTA​TTC​AAG​CCT​CCC​AT
Human CXCL10	TGG​CAT​TCA​AGG​AGT​ACC​TC
	TTG​TAG​CAA​TGA​TCT​CAA​CAC​G
Human IL-6	ACT​CAC​CTC​TTC​AGA​ACG​AAT​TG
	CCA​TCT​TTG​GAA​GGT​TCA​GGT​TG
Human GAPDH	CGG​AGT​CAA​CGG​ATT​TGG​TC
	GAC​AAG​CTT​CCC​GTT​CTC​AG
Human Actin	CAT​GTA​CGT​TGC​TAT​CCA​GGC
	CTC​CTT​AAT​GTC​ACG​CAC​GAT

### 2.10 Immunoprecipitation assay

For immunoprecipitation assay, HEK-293T cells were inoculated overnight. Cells were transfected with Flag-IRF3 or Flag-TBKI and HA-STING or HA-IRF3 plasmids for 18 h. After the treatment with GUP for 4 h, cell pellets were collected and resuspended in lysis buffer (50 mM Tris [pH 7.45], 0.5% [v/v] Triton X-100, 150 mM NaCl, and 1% [m/v] containing protease inhibitors. In addition, the supernatant was incubated with M2 (A2220, Sigma) beads for 4 h at 4°C. The samples were washed three times with the lysis buffer, denaturalized by boiling with 1×RIPA loading buffer for 15 min and analyzed by immunoblotting.

### 2.11 STING oligomerization assay

Oligomerization experiments of STING was performed as previously reported (Li S. et al., 2018). In short, the cell lysates were loaded onto the native-PAGE gel using the native sample buffer, and electrophoresed for 50 min at 25 mA, and then analyzed by immunoblotting.

### 2.12 *In vivo* mice model of STING agonist (DMXAA) and CLP mice model

C57BL/6 wild Eighteen 8-week-old C57 mice were randomized and equally divided into three groups and administered vehicle or GUP (400 mg/kg) by gavage for five consecutive days. According to the relevant research reports and the exploration in the early stage of the experiment, we finally determined the dose selection of GUP (400 mg/kg) for subsequent animal experiments (Huang C. et al., 2022; Wu et al., 2022). On the sixth day, the mice were injected intraperitoneally injection (i.p.) with DMXAA (25 mg/mL). DMXAA dose selection as previously described (Khan et al., 2021). After 4 h, the mice were euthanized, intraperitoneal lavage liquid and serum were collected and DMXAA-induced cytokine levels were measured. GUP was dissolved in 0.9% saline and then administered to mice by gavage. DMXAA was dissolved in 1:1 with PEG300: normal saline.

CLP sepsis was induced with C57BL/6 female mice (8 weeks old) as mentioned earlier (n = 6 mice per group, 18 in total) (Zeng et al., 2017). In short, the mice were anesthetized and the abdominal surgical area was sterilized. Next, a minor incision was created in the middle of the abdomen, and the cecum was brought outside and secured with silk by tying it. We punctured the ligated portion of the cecum with a 22-gauge needle twice before returning it to the abdominal cavity. Finally, the abdomen was sutured into two layers and mice were immediately injected with saline at 50 mg/kg to prevent shock. Sham surgeries involve exteriorizing the cecum and reintroducing it into the abdominal cavity without ligating or puncturing it. Immediately after the operation, Ringer’s solution was injected subcutaneously into the mice.

After 72 h, the mice were executed through cervical dislocation and lung, heart, liver and kidney tissues were taken. One of the lung tissues was paraffin-embedded, followed by hematoxylin-eosin (H&E) staining using standard techniques. Heart, liver and kidney tissues were homogenized and then RNA was extracted for qPCR.

### 2.13 Statistical analysis

The software GraphPad Prism eight and SPSS statistics 26.0 were utilized for the analysis of all experimental data. The statistical evaluation was conducted by an unpaired Student’s t-test between two groups or one-way ANOVA with Dunnett’s *post hoc* test was used for multi groups. The results were shown as Mean ± Standard error of mean (SEM), and *p* values were presented by *p < 0.05, **p < 0.01 and ***p < 0.001.

## 3 Result

### 3.1 The quality control of GUP and cytotoxic effects of GUP

 In order to clarify the evidence for the chemical characterization of GUP, ion chromatography was utilized to determine the monosaccharide composition. The monosaccharide component of GUP was measured by comparison with the reservation time and chromatograph peaks of standard monosaccharides including glucose, galactose and glucuronide acid. Data revealed that the molar ratio of glucose, glucuronide acid and galactose in the GUP sample was 0.902, 0.056, 0.043 respectively (Supplementary Figures S1A, B). The structural features of the GUP were analyzed by FT-IR and the IR spectra were scanned in the range of 4,000-400 cm^-1^ and the results are shown in Supplementary Figure S1C. The infrared spectrogram shows the typical characteristics of GUP (Mowat and Agace, 2014; Nosalova et al., 2013), from which it can be seen that the broad and strong absorption peak at 3,388.50 cm^-1^ is the O-H stretching vibration of the sugar, the smaller absorption peak at 2,929.65 cm^-1^ is the stretching vibration of the methyl or methylenecyclohexane C-H, and the peaks between 1,400∼1,200 cm^-1^ are supposed to be the sugar's C-H variable angle vibration, from the above it can be determined that GUP is polysaccharide. The peaks at 1,616.80 and 1,416.30 cm^-1^ are caused by the asymmetric and symmetric telescopic vibration of C=O in saccharide (Pastorino et al., 2018; Ren et al., 2022a; Ren et al., 2022b), which indicates that GUP contains saccharide, and the results of the determination of the composition of monosaccharides containing glucuronide results are consistent with the results. The peaks between 1,200∼1,000 cm^-1^ between 1,200 and 1,000 cm^-1^ is the characteristic absorption peak of pyranose (Rizzato et al., 2017; Schulte et al., 2013). The above infrared test results indicate that GUP is acidic polysaccharides and contain pyranose rings. Methylation with GC–MS is widely used to determine the linkage types and ratios of polysaccharides. The methylated polysaccharide was analyzed by GC–MS, and the results were summarized in Table 3, which showed that GUP contained: t-linked Glcp and few t-linked Galp, 1,2-linked Glcp, 1,4-linked Glcp, 1,6- linked Glcp, 1,6-linked Galp. We examined the cytotoxicity of GUP in BMDMs and THP-1 cells to learn more about its impact on cGAS-STING pathway activation. A different concentration of GUP was applied to THP-1 and BMDMs cells for 12 h. As shown (Supplementary Figure S2A, B), GUP had no cytotoxicity at concentrations below 3 mg/mL in both cells. Subsequent experiments were carried out at non-toxic doses.

### 3.2 GUP suppresses the activation of HT-DNA-induced cGAS-STING pathway

**FIGURE 1:**
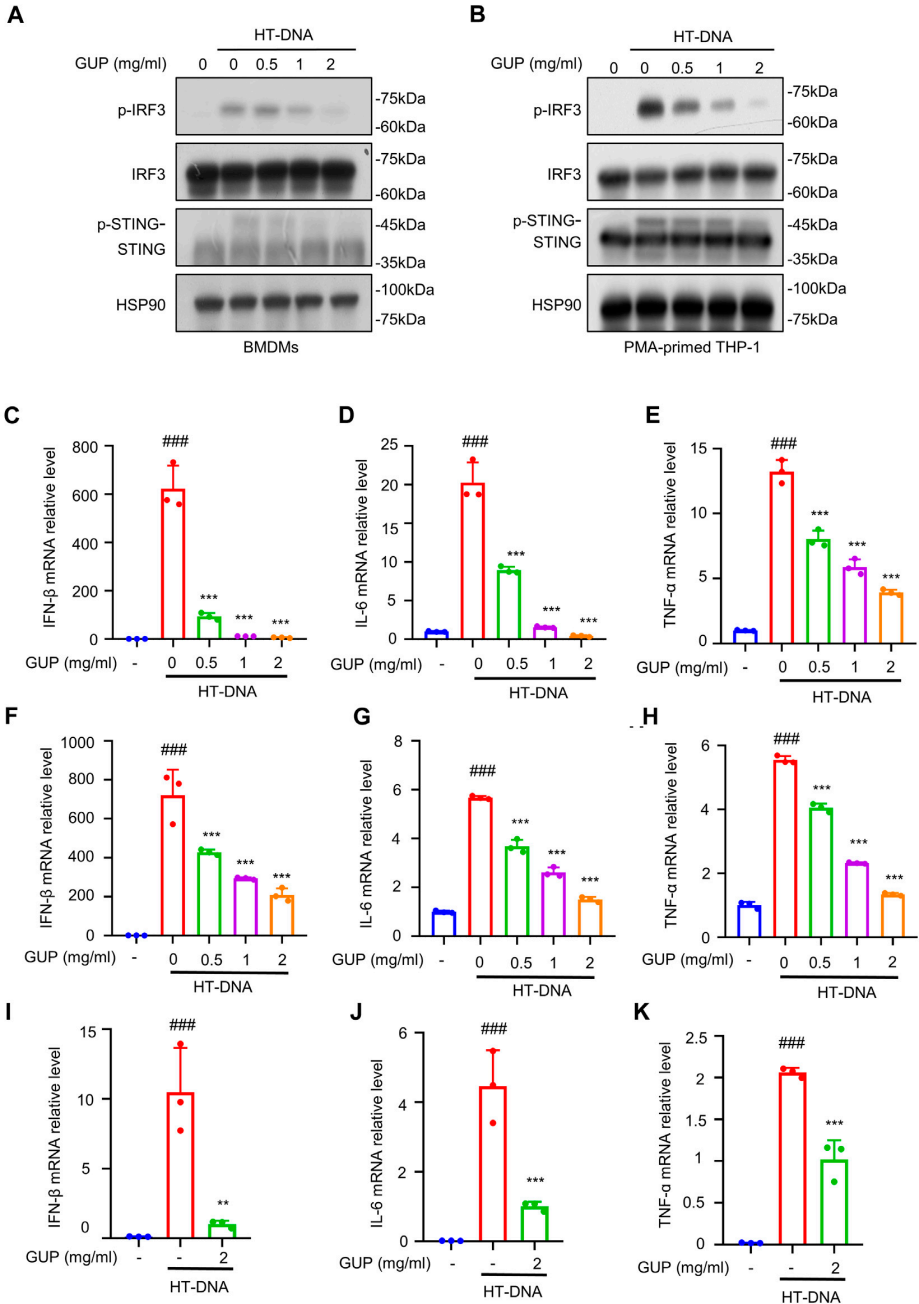
GUP inhibits the activation of HT-DNA-induced cGAS-STING pathway. **(A)** BMDMs and **(B)** THP-1 cells were pretreated with the different concentrations of GUP and then transfected with HT-DNA. Western blotting was conducted to determine phosphorylated p-IRF3, IRF3, p-STING and STING protein levels. Following the steps as above **(A,B)**, HT-DNA was transfected with for 4 h. The mRNA expression of IFN-β, TNF-α and IL-6 was measured by quantitative PCR assay in **(C–E)** BMDMs cells and **(F–H)** THP-1 cells. **(I–K)** Human PBMCs. Relative changes were quantified using the ΔΔCt method. The groups were compared using one-way ANOVA statistics. The results were shown as Mean ± SEM from three biological replicates. **p < 0.01 and ***p < 0.001 vs the stimulated group.

Researches have shown that HT-DNA enters the cytoplasm through transfection and can be sensed by cGAS and then activate STING (Chanut and Petrilli, 2019). Monocytes and macrophages, as secretory cells, are essential for the regulation of the immune response and the development of inflammation (Johnston, 1988). So, we used BMDMs and THP-1 cells to explore whether GUP affected the cGAS-STING pathway. Phosphorylation of IRF3 is a pivotal downstream signaling event in the cGAS-STING pathway. Using immunoblotting analysis to detect related proteins, we found that GUP treatment suppressed the phosphorylation of IRF3 induced by HT-DNA (Figures 1A, B). Meanwhile, we evaluated the effect of GUP on mRNA expression of downstream genes related to this pathway, including IFN-β, IL-6 and TNF-α. Surprisingly, GUP inhibited the mRNA expression of these genes in BMDMs (Figures 1C–E) and THP-1 (Figures 1F–H) cells. More importantly, GUP also inhibited HT-DNA-induced mRNA expression of IFN-β, IL-6 and TNF-α in human PBMCs (Figures 1I–K). In conclusion, these data demonstrate that GUP can suppress the activation of HT-DNA-induced cGAS-STING pathway *in vitro*.

### 3.3 GUP inhibits multiple stimulus-induced activation of the cGAS-STING pathway

**FIGURE 2:**
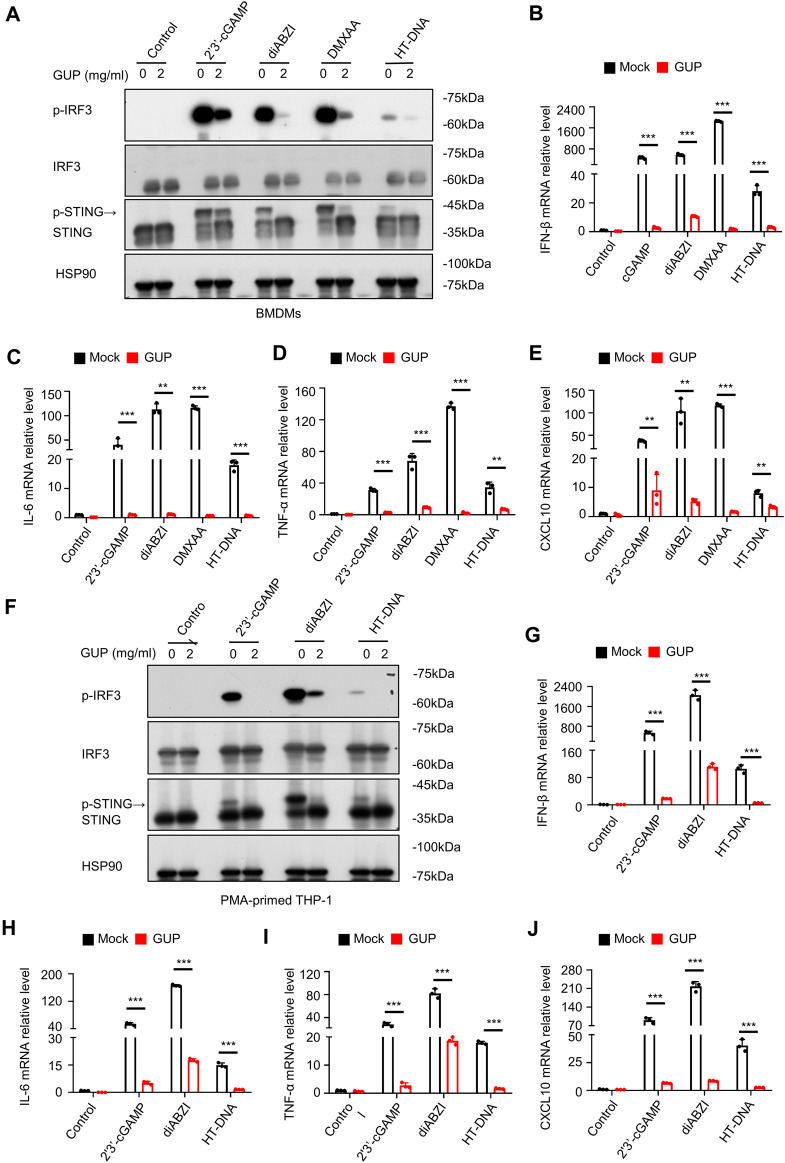
GUP inhibits multiple agonist-induced activation of the cGAS-STING pathway. **(A)** BMDMs and **(F)**THP-1 cells were pretreated with GUP (2 mg/mL) and then transfected with 2′3′-cGAMP and HT-DNA, and addition of diABZI and DMXAA. Phosphorylated expression of p-IRF3, IRF3, p-STING and STING was determined by Western blotting. The mRNA expression of IFN-β, IL-6, TNF-α and CXCL10 was measured by quantitative PCR assay in **(B–E)** BMDMs cells and **(G–J)** THP-1 cells. Following the steps above **(A,F)**, 2′3′-cGAMP and HT-DNA was transfected, and diABZI and DMXAA were added for 4 h. Statistical comparisons between groups were performed using unpaired two-tailed Student's t tests. The results were shown as Mean ± SEM from three biological replicates, and *p*-value were presented by **p < 0.01 and ***p < 0.001 mock group (no administration) *versus* group (administration).

We next investigated GUP affects the activation of the cGAS-STING pathway induced by multiple STING agonists. Some natural or enzymatically synthesized STING agonists, such as 2′3′-cGAMP, diABZI and DMXAA, can also induce the release of type Ⅰ IFNs and other pro-inflammatory factors (Jin et al., 2011; Messaoud-Nacer et al., 2022). We further study found that GUP inhibited the phosphorylation of IRF3 induced by various typical STING stimulators (HT-DNA, 2′3′-cGAMP, diABZI and DMXAA) in BMDMs (Figure 2A) and THP-1 (Figure 2F) cells. In addition, to further validate the above results, we also performed qPCR on its related downstream genes. Interestingly, GUP markedly inhibited the mRNA expression of IFN-β, IL-6, TNF-α and interferon-stimulated genes (CXCL10) activated by multiple STING stimulators in BMDMs (Figures 2B–E) and THP-1 (Figures 2G–J) cells. All in all, these results strongly demonstrate that GUP has an essential regulatory effect in cGAS-STING pathway activation triggered by multiple stimulus.

### 3.4 GUP disrupts nuclear translocation of IRF3

**FIGURE 3:**
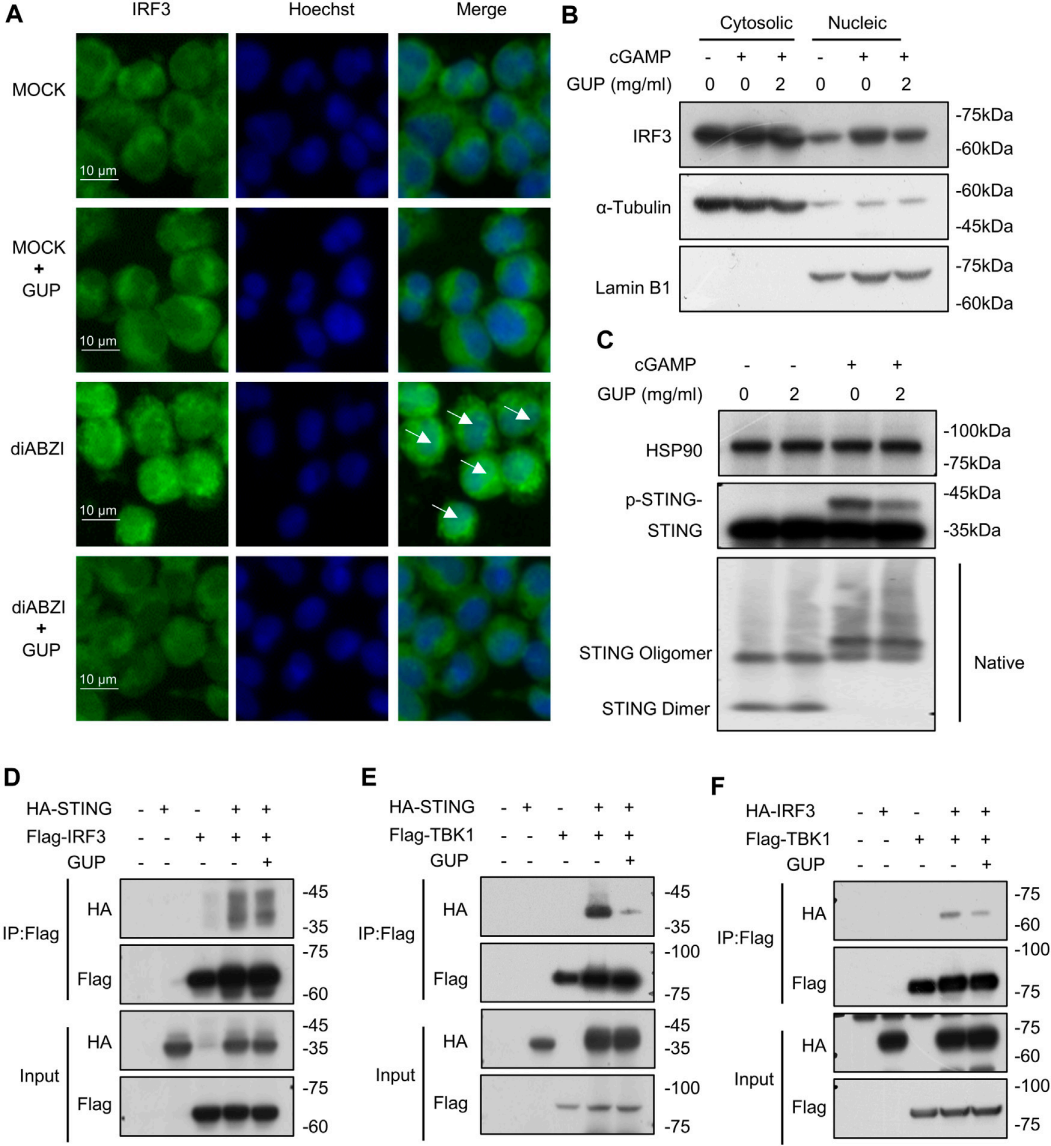
GUP disrupts nuclear translocation of IRF3 and inhibits STING-TBK1 and TBK1-IRF3 interactions. **(A)** THP-1cells were treated with GUP for 1 h, followed by stimulation with diABZI for 2 h and stained for IRF3. The blue Hoechst (nucleus) image is superimposed on the green IRF3 image. At 20 original magnification, pictures were shot for each circumstance. Scale bars represent 10 μm. **(B)** 2′3′-cGAMP was transfected into THP-1 cells for 2 h after GUP treatment for 1 h. Protein expression in cytosolic and nuclear lysate of THP-1 cells treated with GUP were analyzed by Western blot to determine the cellular distribution of IRF3. **(C)** GUP was pretreated for 1 h in BMDMs, and then 2′3′-cGAMP was transfected for 1 h in the cells. Collection of lysates for immunoblotting to detect phosphorylation and oligomerization of STING. **(D)** Flag-TBK1 or **(E)** Flag-IRF3 plasmids was co-transfected with HA-STING plasmids. **(F)** Flag-TBK1 plasmids was co-transfected with HA-IRF3 plasmids. HEK-293T cells were treated with GUP for 4 h after 18 h of transfection. The anti-DYKDDDDK (Flag) affinity gel agarose beads were used for immunoprecipitation. Finally, we performed immunoblot analysis.

Nuclear translocation is an important function in transcription. IRF3 is phosphorylated to form a dimer that translocate to the nucleus and thereby regulates the expression of type I IFNs (Zhang et al., 2019). Here, we explored whether GUP could inhibit the nuclear translocation of IRF3, thereby suppressing transcriptional activity. As shown in the picture (Figure 3B), GUP inhibited the nuclear accumulation of IRF3. To verify these results, we performed an immunofluorescence assay. Our results showed (Figure 3A), GUP significantly reduces diABZI induced nuclear aggregation of IRF3. The present study demonstrated that GUP blocked IRF3 transcriptional activity and thereby suppressed the expression of type I IFNs.

### 3.5 GUP suppresses the activation of cGAS-STING pathway by inhibiting STING-TBK1 and TBK1-IRF3 interactions

Next, we aim to explore the mechanisms by which GUP influences cGAS-STING pathway activation. Our previous research demonstrated that GUP could suppress the phosphorylation and nuclear translocation of IRF3, a key downstream signaling event in this signaling pathway. Therefore, we will further explore the role of GUP on the upstream of this pathway. Studies have shown that cGAMP causes a conformational change in STING, resulting in oligomerization of STING and subsequently recruits TBK1 to activate downstream pathways. However, our results indicated that GUP did not inhibit 2′3′-cGAMP-induced STING oligomerization (Figure 3C). STING is a scaffold protein that, upon binding to CDNs, recruits downstream signaling proteins (Li S. et al., 2018). After STING recruits TBK1, IRF-3 transcription factors are subsequently recruited to the signaling complex and phosphorylated by TBK1, which then regulates downstream pathways (Zhao et al., 2019; Ren Y. et al., 2022). Thus, binding between STING, TBK1 and IRF3 is necessary for activating cGAS-STING pathway. To this end, we examined whether GUP inhibited the interaction between three key proteins in this pathway, STING, TBK1 and IRF3. Notably, our results showed that in exogenous scenarios, GUP inhibited STING interactions with TBK1 (Figure 3D) and TBK1 interactions with IRF3 (Figure 3E), but not STING-IRF3 interactions (Figure 3F). In conclusion, these results suggest that GUP inhibits the activation of the cGAS-STING pathway by inhibiting the interaction of STING with TBK1 and TBK1 with IRF3.

### 3.6 GUP inhibits the activation of cGAS-STING *in vivo*

**FIGURE 4:**
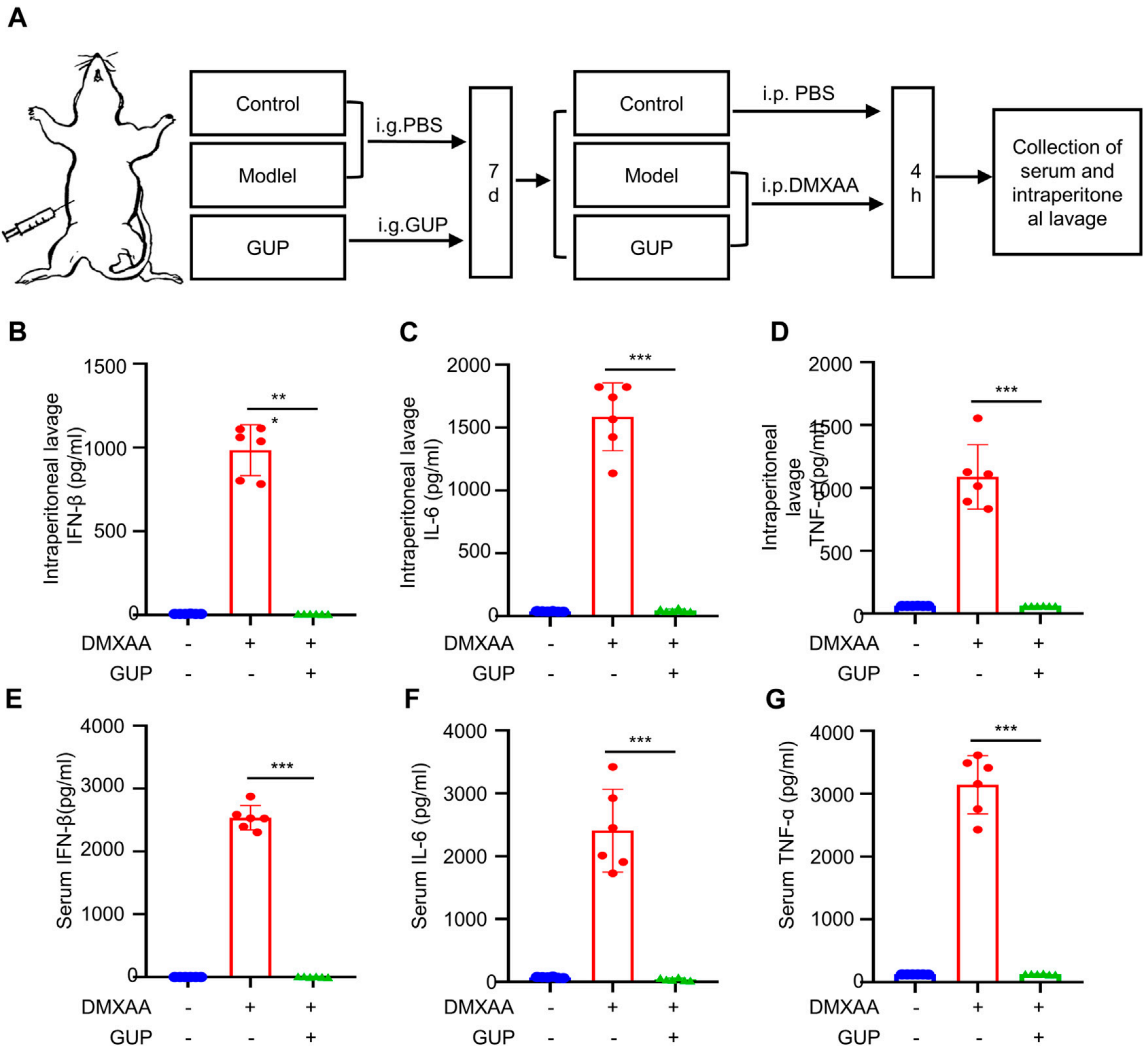
GUP suppresses the activation of cGAS-STING *in vivo*. *In vivo* experiments of DMXAA-induced activation of STING and its downstream signaling pathways *in vivo* **(A)**, the levels of IFN-β, IL-6 and TNF-α respectively in the **(B–D)** intraperitoneal lavage and **(E–G)** serum of mice after 4 h of DMXAA induction were determined by ELISA. Data were displayed by Mean ± SEM (n = 6 mice per group). The groups were compared using one-way ANOVA statistics. ^###^ *p* < 0.001, ***p < 0.001 *versus* the DMXAA group. (n = 6 mice per group).

Our next step was to investigate how GUP affects the activation of the cGAS-STING pathway *in vivo*. DMXAA, a mouse interferon gene (STING) stimulator (Xu et al., 2021), was also a potent inducer of type I IFNs and inflammatory cytokines, so we chose DMXAA for our *in vivo* study. Mouse were given GUP (400 mg/kg/day) for 5 days and DMXAA was injected intraperitoneally on the sixth day. After 4 h, serum and intraperitoneal lavage liquid were gathered from the mouse and the expression of IFN-β, IL-6 and TNF-α in the serum and intraperitoneal lavage fluid were measured by Elisa kits. Our results showed that DMXAA stimulation raised the expression of IFN-β, IL-6 and TNF-α in the intraperitoneal lavage liquid (Figures 4B–D) and serum (Figures 4E–G) of mouse. Notably, GUP markedly decreased DMXAA-mediated expression of IFN-β, IL-6 and TNF-α. Thus, GUP can inhibit the activation of the cGAS-STING pathway *in vivo*.

### 3.7 GUP alleviates CLP through regulating cGAS-STING pathway

**FIGURE 5:**
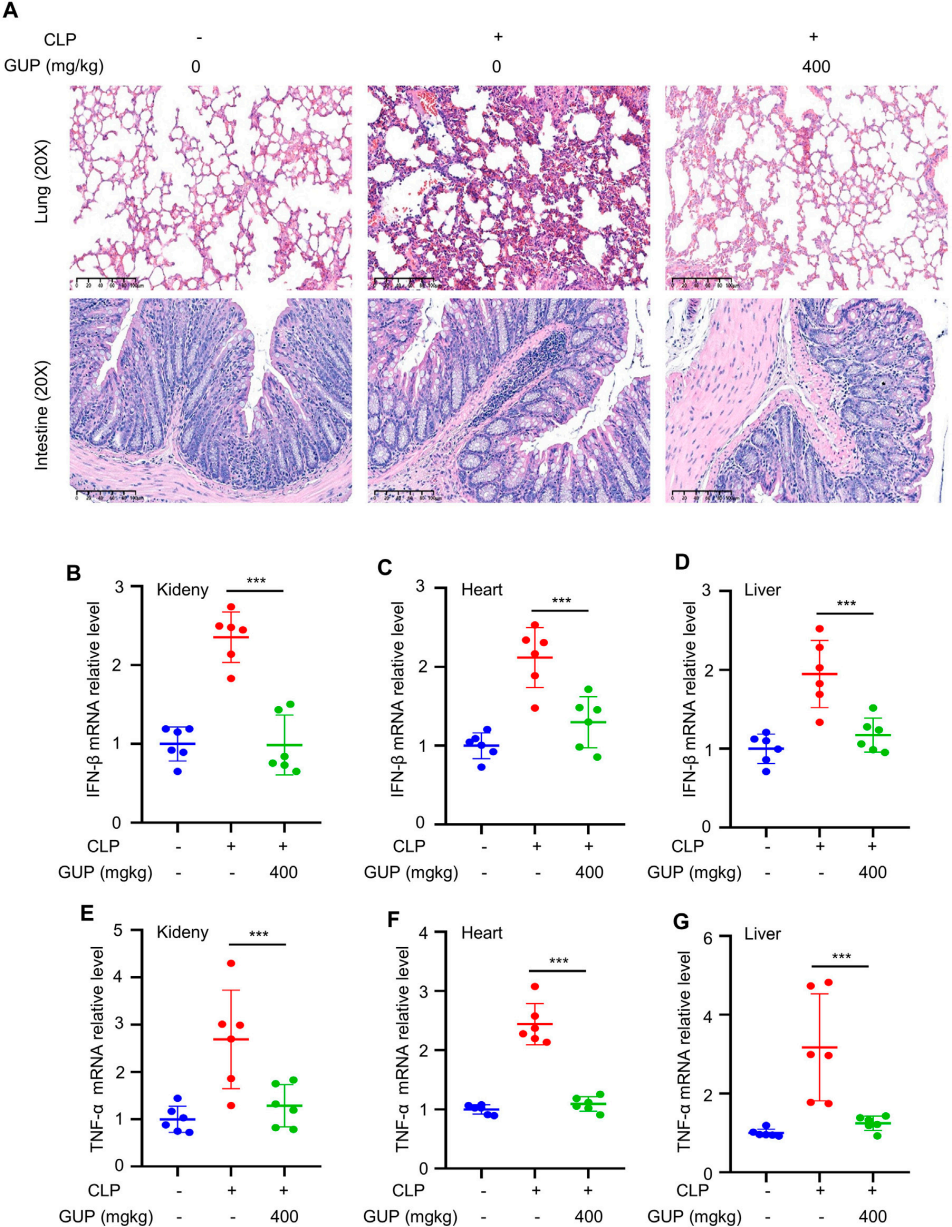
GUP alleviated CLP through regulating cGAS-STING signaling pathway. **(A)** H&E staining of lung and intestine tissues. Scale bar: 100 μm. (*n* = 6 mice per group). **(B–D)** IFN-β and **(E–G)** TNF-α cytokine mRNA in Kidney, Heart and Liver. One-way ANOVA statistics were used to compare the groups. The results were shown as Mean ± SEM. ^##^ *p* < 0.01 and ^###^ *p* < 0.001 means compared to the control group; **p < 0.01 and ***p < 0.001 *versus* the CLP group (n = 6 mice per group).

Study showed significant activation of cGAS-STING pathway in Cecal Ligation and Puncture (CLP)-induced sepsis (Li J. et al., 2022). In recent years, various models of sepsis have been studied, and CLP model is the most associated animal model for clinical sepsis (Buras et al., 2005; Dejager et al., 2011). To determine the effect of GUP on sepsis, CLP was conducted following the previous description (Zeng et al., 2017). GUP (400 mg/kg/day) was treated to mouse for 5 days before CLP and repeated oral administration to mouse at 12, 24, 48 and 72 h after CLP. Through the analysis of H&E staining of lung and intestinal tissues of mouse, we found that CLP-induced lung and intestine increased leukocyte infiltration and significant accumulation of inflammatory cells in the alveolar space, in accordance with previous researches (Zeng et al., 2017). Our results showed that GUP reduces damage to lung and intestinal tissues (Figure 5A). Furthermore, in CLP-induced heart, liver and kidney tissues, the mRNA expression of IFN-β and TNF-α was increased at 72 h, whereas GUP decreased the mRNA expression of IFN-β (Figures 5B–D) and TNF-α (Figures 5E–G). In general, these data suggest a protective effect of GUP against CLP sepsis. Combining the results of previous experiments and the action of the cGAS-STING pathway in the inflammatory response (Decout et al., 2021), we speculate that GUP may exert its anti-inflammatory effects by down-regulating the cGAS-STING pathway.

## 4 Discussion

Studies have indicated that the cGAS-STING pathway is significantly expressed in a variety of inflammatory diseases, such as sepsis (Li J. et al., 2022), kidney injury (Maekawa et al., 2019) and lung injury (Huang R. et al., 2022). Sepsis is an acute disease caused by an infection or trauma resulting in a systemic inflammatory response caused by microbial invasion, resulting in a dysregulated host response leading to organ dysfunction (Evans, 2018). The search for an inhibitor targeting the regulation of the cGAS-STING pathway is a promising line of research for the treatment of sepsis. *Glycyrrhiza uralensis* is a traditional medicinal plant that has demonstrated antiviral and anti-inflammatory activity (Wang et al., 2015; Yang et al., 2017; Babu et al., 2023). This article reports that GUP, the main active constituent of *G. uralensis*, suppressed the activation of cGAS-STING both *in vitro* and *in vivo*, revealing the effect of GUP in regulating cGAS-STING-mediated sepsis.

It has been previously reported that GUP have antioxidation, antibacterial and anti-tumor activities, but little research has been done on anti-inflammatory activities (Lian et al., 2018; Hao et al., 2020). Zhao et al. found that GUP alleviated the symptoms of DSS-induced UC mice by suppressing the expression of IL-1, IL-6 and TNF-α in serum (Huang C. et al., 2022). Although its anti-inflammatory effects have been studied to some extent, its exact mechanism of action and targets are not yet clear. Our research showed that GUP inhibited the phosphorylation of IRF3 and the expression of mRNA of related downstream gene to the cGAS-STING signaling pathway, such as IFN-β, IL-6, TNF-α and CXCL10 induced by various canonical cGAS- STING agonists (HT-DNA, 2′3′- cGAMP, diABZI and DMXAA). This indicates that GUP can influence the aberrant activation of the cGAS-STING pathway and is a broad-spectrum inhibitor of the cGAS-STING pathway with inhibition of the pathway *in vitro*. Previous research has revealed that the STING agonist DMXAA causes systemic inflammatory responses and shock-like symptoms in mice (Brault et al., 2018). It is noteworthy that GUP markedly suppressed the release of systemic cytokine in DMXAA-induced mice. This results showed that GUP can suppress the activation of the cGAS-STING pathway *in vivo*. However, DMXA has not been shown to be an agonist for human STING, so it remains to be investigated whether GUP can effectively inhibit the activation of the cGAS-STING pathway in humans.

STING is activated by cGAMP and recruits TBK1 and IRF3. IRF3 forms a complex with STING and TBK1 and is phosphorylated by TBK1, thereby regulating downstream gene expression (Ghosh et al., 2021). Studies demonstrate that the interaction between three proteins, STING, TBK1 and IRF3 are important for the activation of the cGAS-STING pathway (Li S. et al., 2018; Ghosh et al., 2021; Ren Y. et al., 2022). In previous study, Licorice flavonoids were indicated to have a good therapeutic effect on acute lung injury induced by LPS, which may act by acting upstream of the cGAS-STING pathway and reducing the synthesis of cGAMP. Licorice extract, on the other hand, can treat MCD-induced NASH mice, and its mechanism of action is to affect the oligomerization of STING. Glabridin and Licochalcone B, as the main active ingredients in licorice, showed promising ameliorative effects on autoimmune diseases contributed to *Trex1* knockout, and their mechanism of action may be related to affecting the binding of IRF3 with STING. Our results indicate that GUP inhibits the cGAS-STING pathway by affecting STING-TBK1 and TBK1-IRF3 interactions. This indicates that although different components of *Glycyrrhiza glabra* have inhibitory effects on the cGAS-STING pathway, the exact mechanism of action is not the same, which also well explains the “multi-component, multi-pathway, multi-target” action characteristics of traditional Chinese medicine. Our study also undoubtedly further enriches the scientific connotation of licorice in “clearing heat and removing toxins”. However, it is precisely because gup contains various monomeric components that its direct action and targeting of proteins need to be further investigated.

Sepsis is a systemic infectious disease caused by toxins from bacteria, fungi or other microorganisms. Research indicates that there is a strong correlation between Intestinal bacteria and sepsis (Buffie and Pamer, 2013). Intestinal bacteria, which are the basis of intestinal metabolites, can reduce the risk of sepsis by regulating beneficial bacteria in the gut such as Bifidobacteria and Lactobacilli to promote the integrity of the intestinal mucosal barrier and reduce the number of harmful bacteria (Mowat and Agace, 2014; Chen Y. et al., 2021). At the same time, changes in intestinal bacteria may also contribute to abnormal activation of the immune system, leading to an excessive inflammatory response, which in turn exacerbates sepsis (Wang et al., 2022). Improving intestinal flora is an effective treatment for sepsis, but finding a precursor compound that is effective in treating sepsis is more responsive to clinical needs. Our ultimate aim is to assess the therapeutic potential of GUP for CLP sepsis.

Walker’s group demonstrated for the first time that *STING* knockout mice were protected from CLP induced sepsis (Heipertz et al., 2017). Li’s laboratory further demonstrated increased cGAS-STING pathway in human sepsis and discovered that blocking cGAS-STING pathway prevented sepsis-associated acute liver injury (Li J. et al., 2022). These data confirm the importance of the cGAS-STING pathway in the development of sepsis. The release of inflammatory cytokines often leads to sepsis and suppression of inflammation is a strategy for treating sepsis (Schulte et al., 2013). In previous studies, type I IFNs is an essential cytokine that promotes and regulates immune and inflammatory responses, and its inappropriate expression leads to the death of various cells, which is essential for the septicemic response (González-Navajas et al., 2012; Li Y. et al., 2018). TNF-α is a key immune system regulator whose overexpression may contribute to the development of inflammatory disorders (Dinarello, 2000). Our results showed that CLP mice showed dysfunction of the heart, liver and kidney, such as increased release of IFN-β and TNF-α, which was in accordance with previous findings (Zeng et al., 2017). H&E staining of the lung and intestine tissues also showed some degree of damage to the lung tissue. Surprisingly, the GUP treatment improved these symptoms. In view of the above results, we hypothesize that the ameliorative effect of GUP on CLP sepsis may act by mediating immune-related pathways, especially the cGAS-STING pathway. However, whether GUP has a protective effect against other cGAS-STING-mediated inflammatory diseases needs to be further explored.

## 5 Conclusion

Our study identifies a potent inhibitor of the cGAS-STING pathway. GUP inhibited the activation of the cGAS-STING pathway *in vitro* and *in vivo*, reducing the expression of related downstream gene. Mechanistically, GUP inhibited the cGAS-STING pathway by inhibiting the interaction of STING with TBK1 and TBK1 with IRF3. At the same time, it also provides a potential therapeutic agent for cGAS-STING pathway-induced related inflammatory diseases, particularly sepsis.

## Data Availability

The original contributions presented in the study are included in the article/Supplementary Material, further inquiries can be directed to the corresponding authors.
