# Supplementary material for: Glycyrrhiza uralensis polysaccharides ameliorates cecal ligation and puncture-induced sepsis by inhibiting the cGAS-STING signaling pathway
Source: Front Pharmacol. 2024 Jun 5;15:1374179. doi: 10.3389/fphar.2024.1374179 (PMC11188434; doi:10.3389/fphar.2024.1374179)
Supplement: Supplementary file 1 [file DataSheet1.zip › Image 1.pdf]

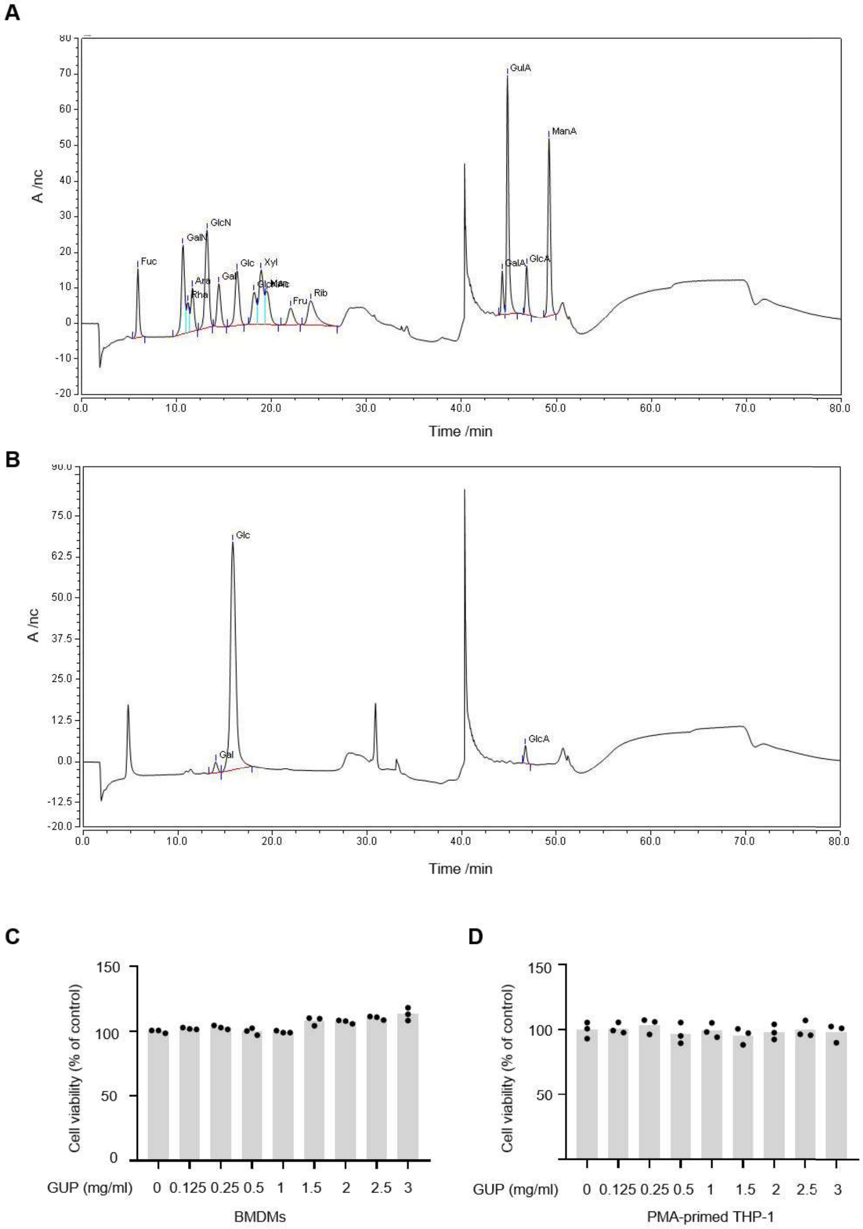

Supplementary Figure 1 Content determination of monosaccharide in GUP and cytotoxicity of GUP.

(a) Monosaccharide composition of mixed monosaccharide standards.

(b) The polysaccharide extract from G. uralensis. The retention time of the peak is respectively, galactose (14.000min), glucose (15.800min), glucuronic acid (46.734min).

(c) BMDMs and (d) THP-1 cells were treated with different concentrations of GUP for 12 h. Cell proliferation and viability were detected by CCK-8 assay (*n* =3).
