# Supplementary figures and images for: Glycyrrhiza uralensis polysaccharides ameliorates cecal ligation and puncture-induced sepsis by inhibiting the cGAS-STING signaling pathway
Source: Front Pharmacol. 2024 Jun 5;15:1374179. doi: 10.3389/fphar.2024.1374179 (PMC11188434; doi:10.3389/fphar.2024.1374179)

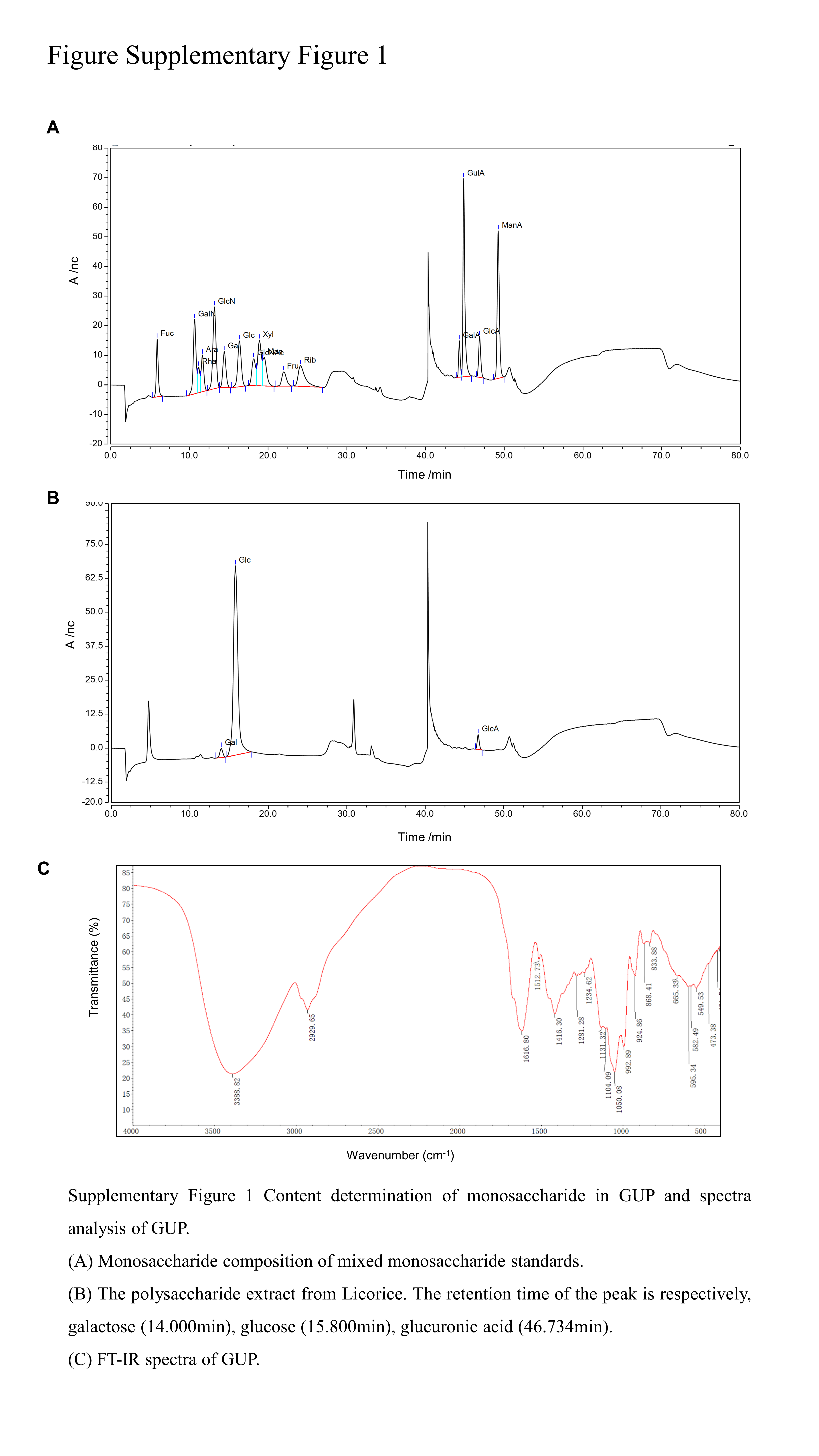

Supplement: Supplementary file 1 [file DataSheet1.zip › Image 1.tif]

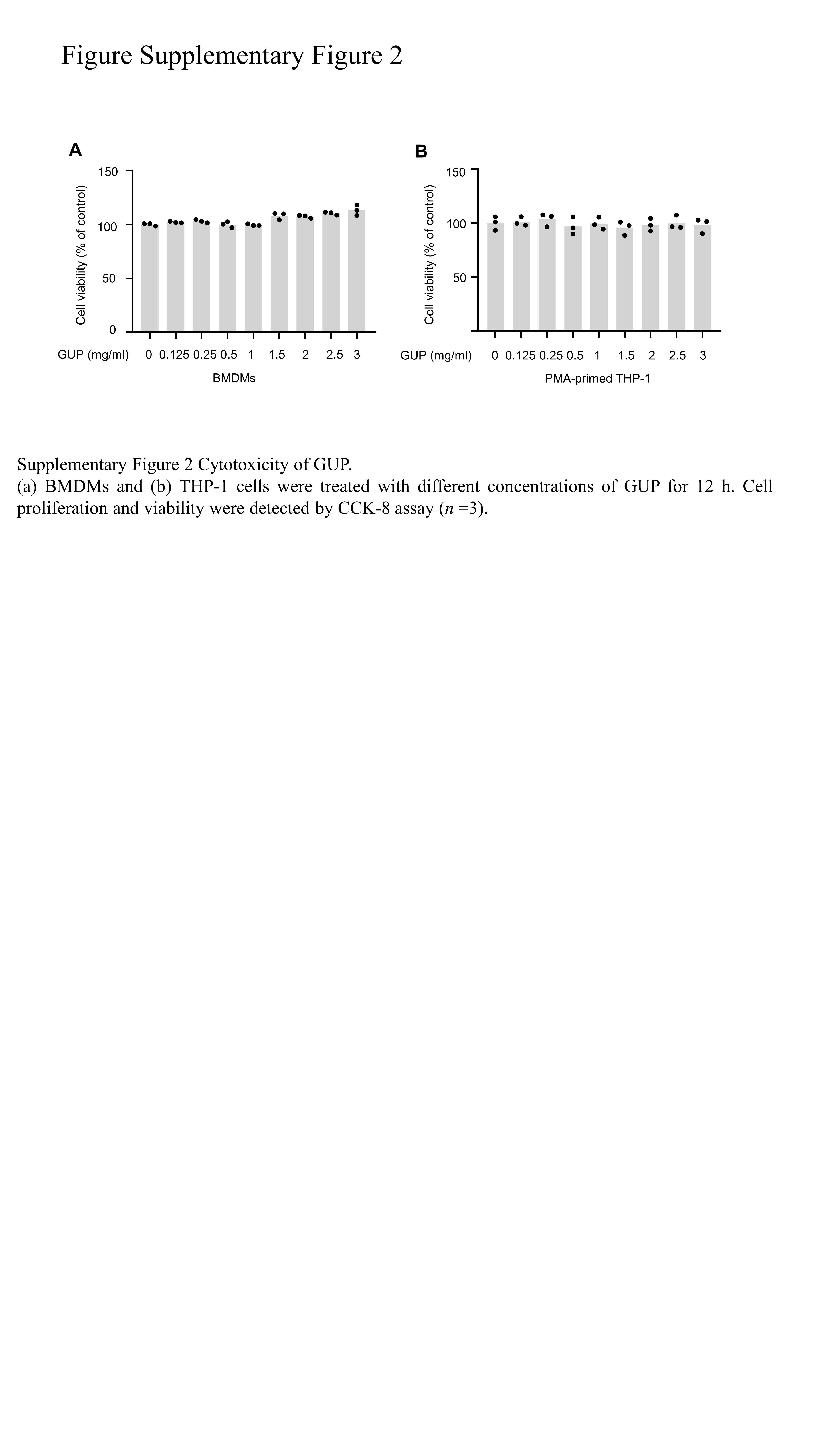

Supplement: Supplementary file 1 [file DataSheet1.zip › Image 2.tif]

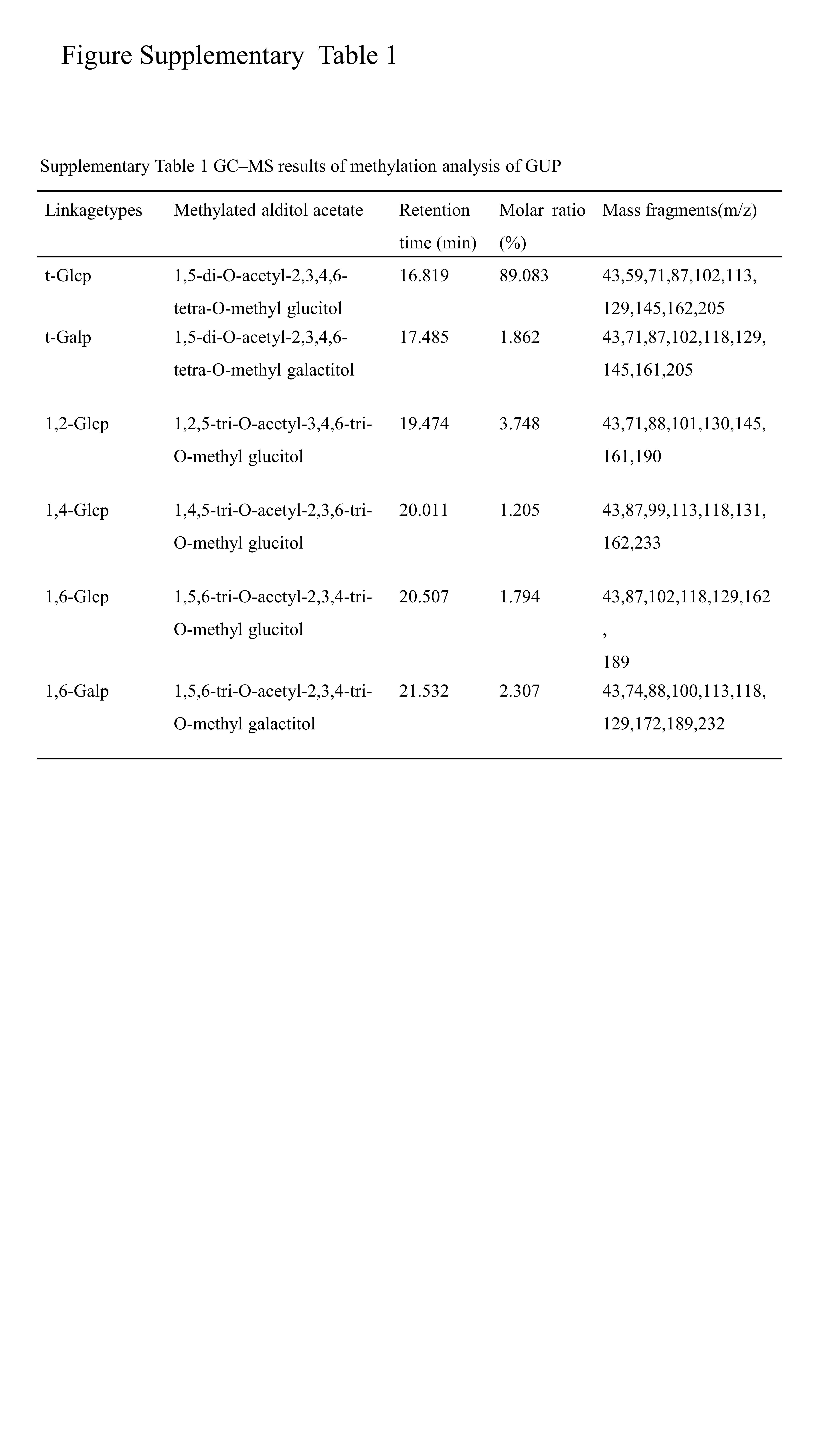

Supplement: Supplementary file 1 [file DataSheet1.zip › Image 3.tif]
